# Bridging medical education and public health: a pedagogical model to develop future physicians’ capacity in audience-tailored health communication

**DOI:** 10.3389/fpubh.2026.1792396

**Published:** 2026-03-04

**Authors:** Minling Liu, Xiaocong Mo, Tingwei Li, Huiru Dai, Shuo Fang

**Affiliations:** Department of Oncology, The Seventh Affiliated Hospital of Sun Yat-sen University, Shenzhen, Guangdong, China

**Keywords:** A-SPIRE teaching model, medical education innovation, medical students, public health education and health promotion, science communication

## Abstract

**Objective:**

Effective health communication is a cornerstone of public health promotion. This study aimed to develop and evaluate a pedagogical model to equip future physicians with essential skills in audience-tailored science communication, thereby contributing to the public health workforce.

**Methods:**

The study involved 79 undergraduate students enrolled in the elective science communication course. The newly proposed A-SPIRE model (Anchoring, Searching, Processing, Integrating, Realizing, Evaluating) was applied in a teaching unit on “Precision Science Communication.” A pre-post design was used, with data collected via questionnaires, knowledge tests, and analysis of student-created communication works.

**Results:**

Students’ understanding of precision science communication concepts and recognition of its importance significantly improved post-course (*P* < 0.05). The overall distribution of knowledge test scores shifted upward (*P* < 0.05). The student-created works effectively addressed the needs and characteristics of different target populations, demonstrating strong relevance, targeting, and effectiveness. The student-created works demonstrated a strong ability to address the specific needs and characteristics of different target populations (adolescents, young/middle-aged adults, older adults). 92.6% of students were satisfied with the model, and 96.3% reported improved communication abilities.

**Conclusion:**

The A-SPIRE model effectively enhances medical students’ competency in precision health communication. It represents a feasible and scalable educational strategy to bridge medical training and public health needs by cultivating a future workforce capable of designing and delivering effective, audience-specific health promotion interventions.

## 1 Introduction

The persistent global burden of disease underscores the critical need for effective health promotion strategies, which fundamentally depend on clear, accessible, and persuasive science communication (1–4). A well-equipped healthcare workforce, competent in translating complex medical information for diverse public audiences, is a vital component of national health promotion capacity (5). In China, the science communication stands as a core component of the “Healthy China” strategy (6). Medical students, as future physicians and frontline health messengers, require specific training to move beyond clinical dialogue and engage in public-facing health communication. Their ability to communicate health science effectively directly influences public health by shaping community behaviors and collective well-being. Research suggests that education and training are vital methods for enhancing practical capacity in health promotion (7, 8).

Despite the high willingness to participate in science communication reported among university students (approximately 80–99.52%), a significant gap exists between intention and practice, with only 17–48% having actually engaged in medical science communication practice (9–11). 71.28% attribute their lack of participation to a perceived deficiency in their own science communication abilities. This highlights an urgent need for structured educational interventions.

The health communication course significantly improved social media competence and professionalism (12). While various approaches such as student clubs, campaign-based teams, or integration into clinical rotations have been explored in Chinese medical schools (13–16), a systematic and transferable pedagogical model focusing specifically on audience-tailored communication skills remains underdeveloped. The 2021 *Outline of the National Action Plan for Scientific Literacy* further explicitly requires higher education institutions to promote the development of science communication as a discipline. Moreover, 70.45% of survey respondents believe that medical schools should offer dedicated courses to enhance students’ science communication abilities (17).

Against this backdrop, Sun Yat-sen University launched the undergraduate elective course *Fundamentals and Practice of Medical Science Communication* in 2023. The course aims to systematically impart theoretical knowledge of medical science communication, strengthen practical skills, and foster a sense of responsibility. The teaching team has previously integrated methods such as the BOPPPS model, Outcome-Based Education, and ideological-political education into the curriculum, accumulating preliminary experience (18, 19). However, a medical science communication course demands not only knowledge transfer but also emphasizes the ability to translate knowledge, communication techniques, and public engagement skills, possessing a strong practical dimension. Therefore, there is a pressing need for a teaching model that can both solidify the theoretical foundation and effectively facilitate practical application.

Through two semesters of teaching practice and iteration, our team has gradually developed and refined the A-SPIRE teaching model. This model aims to provide a structured pathway from theoretical understanding to practical creation of tailored health messages. Grounded in experiential learning and constructivist theory, A-SPIRE comprises six iterative stages: Anchoring (setting context and motivation), Searching (active information gathering), Processing (organizing and interpreting information), Integrating (synthesizing knowledge for specific contexts), Realizing (producing a communication artifact), and Evaluating (multi-source reflective assessment).

This study evaluates the applicability and effectiveness of the A-SPIRE model in cultivating medical students’ capacity for precision science communication. We hypothesize that this model will improve students’ cognitive understanding, knowledge acquisition, and practical skills in creating audience-specific health communication materials, thereby offering a valuable framework for public health education within medical curricula.

## 2 Materials and methods

### 2.1 General information

This study involved 79 undergraduate students from Sun Yat-sen University (class of 2022–2024) enrolled in the elective course *Fundamentals and Practice of Medical Science Communication.*

### 2.2 Methods

#### 2.2.1 Course setting

The study focused on the 8th session, “Precision Science Communication and Tailoring to Different Audiences” (October 2025, 135 min total).

#### 2.2.2 Instructional design

**Figure 1:**
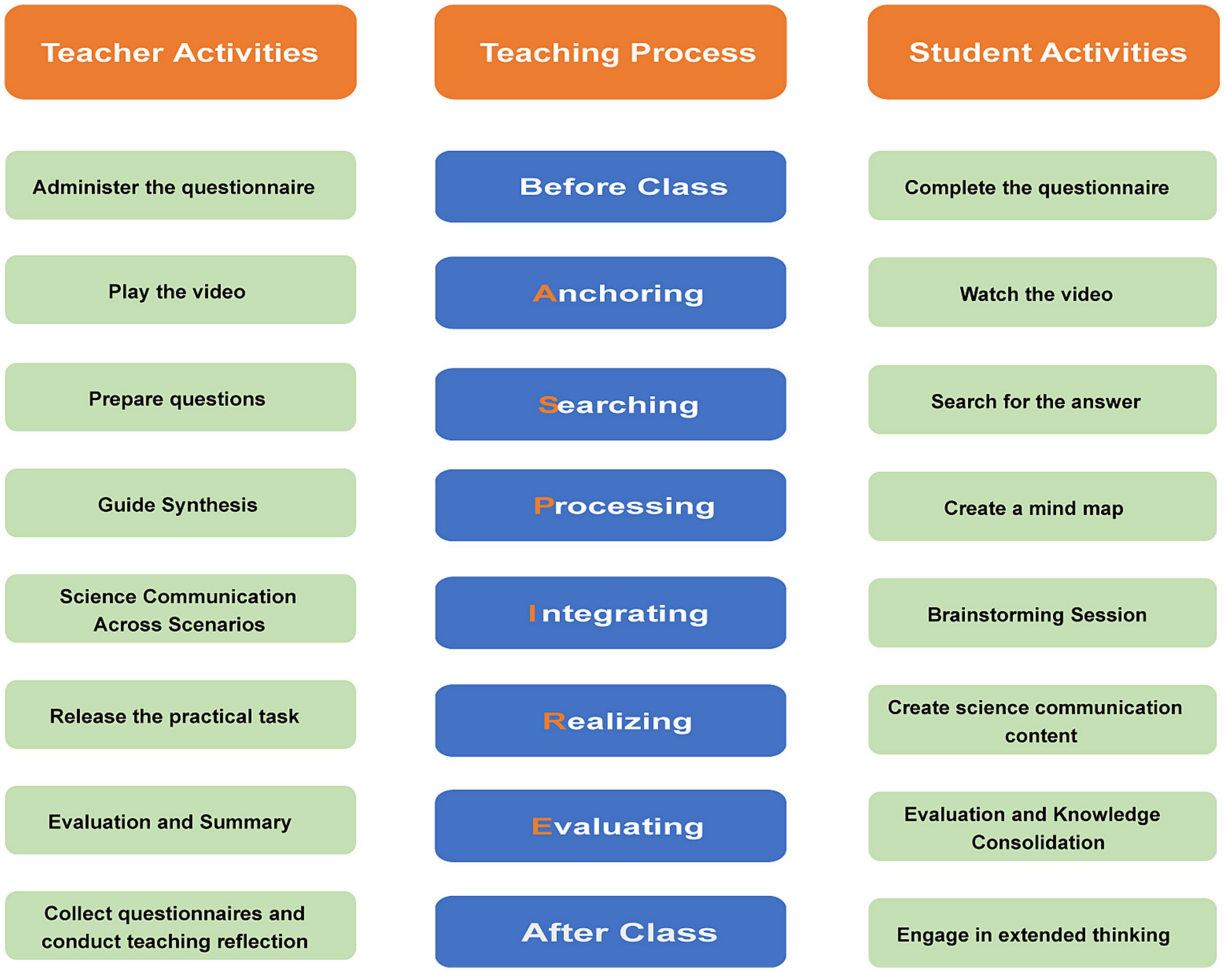
The framework of the “science communication-embedded” instructional model. This figure depicts a dual-track, phased teaching framework designed to cultivate science communication capacity. The central axis outlines the cognitive progression of learners through six stages: Anchoring Phase (10 min): Used film clips and scenarios to introduce the challenge of communicating effectively with different publics. Searching Phase (10 min): Students researched characteristics and needs of assigned demographic groups (adolescents, adults, older adults). Processing Phase (10 min): Students used mind maps to organize the core principles of audience segmentation and tailored messaging, thereby deepening their understanding of precision science communication. Integrating Phase (45 min): Theoretical concepts were applied to plan communication strategies for the three specific demographic groups. Realizing Phase (45 min): Student groups created a precision science communication work (mask design) on “oral health” for their assigned audience. Groups shared their final works in a WeChat group, with explanations specifying the target audience, their relevant characteristics, and the specific needs or challenges addressed. Evaluating Phase (15 min): Multi-dimensional assessment (self, peer, teacher) focusing on entertainment value, relevance, and effectiveness.

The design was divided into three parts. (1) Pre-class Preparation: The instructor distributed a questionnaire to assess students’ prior knowledge and readiness, facilitating instructional planning. Students completed the questionnaire while gaining a preliminary understanding of the session’s content. (2) In-class A-SPIRE Model Application: The session was structured around the six stages of the A-SPIRE model (Figure 1). (3) Post-class Extended Learning: Students completed reflection questions to consolidate and deepen their understanding. The instructor collected feedback for teaching reflection and continuous improvement.

### 2.3 Observation indicators

Questionnaires and test questions were administered using the Wenjuanxing online assessment platform.

#### 2.3.1 Understanding of precision science communication

Pre- and post-course 5-point Likert scale surveys on concept familiarity and perceived importance.

#### 2.3.2 Mastery of precision science communication knowledge

A knowledge test consisting of 6 multiple-choice questions, each worth 10 points for a total of 60 points, was used. The test content focused on core knowledge of precision science communication, specifically including the characteristics, science communication needs, key challenges (“pain points”), and preferred formats for different demographic groups. The same test was administered before and after the intervention for quantitative assessment of the teaching effect.

#### 2.3.3 Practical application ability

Work analysis was employed to examine students’ ability to translate theoretical knowledge into practical outcomes. The primary evaluation criterion was whether the science communication works achieved precision targeting, encompassing four dimensions: entertainment value, relevance, effectiveness, and suitability of format. Research indicates that in health communication, precisely identifying the target audience and conducting detailed user profiling can enhance the persuasiveness of the content and promote health behavior change among the audience (8). (1) Entertainment Value: The ability to accurately capture characteristics of the target demographics to attract their attention and generate willingness to view, i.e., “want to watch.” For example, cultural backgrounds effect the health communication (20). (2) Relevance: Whether the content aligns with the genuine needs of the audience (e.g., dental caries in adolescents, pulpitis in young/middle-aged adults, tooth loss in older adults), ensuring the communicated knowledge has practical value, making the audience feel it is “worth watching.” (3) Effectiveness: The ability to overcome barriers (“pain points”) different groups face in accessing science communication knowledge (e.g., cognitive limitations in adolescents, inertia in taking action among young/middle-aged adults, declining comprehension and memory in older adults), ensuring the knowledge can be accessed by the audience, i.e., “can be seen.” Research finds that humans often exhibit resistance to behavior change (21). (4) Suitability of Format: The ability to select appropriate science communication methods to lower barriers to information reception, enabling efficient knowledge transfer, achieving “easy viewing.” As this classroom practice employed a uniform format (mask creation), suitability of format was not included as an evaluation criterion for this specific exercise.

#### 2.3.4 Students self-assessment

A 5-point Likert scalewas used to survey students on their satisfaction with the teaching model, whether the session helped improve their science communication abilities, whether the session would assist their future science communication practice, and their willingness to attempt precision science communication in the future.

### 2.4 Statistical methods

Cronbach’s alpha coefficient was used to assess the reliability of the questionnaires. Statistical analysis was performed using SPSS software (version 27.0). As the number of participants in the pre- and post-intervention surveys differed, and the groups could not be perfectly matched, they were treated as two independent samples for analysis. Inter-group comparisons were conducted using non-parametric tests (Mann–Whitney U test). A two-sided *P* < 0.05 was considered statistically significant. The normality of test scores was assessed using the Shapiro–Wilk test. The results indicated that both pre- and post-intervention test scores did not follow a normal distribution. Therefore, test scores are described using the median (*M*) and interquartile range (*P*25, *P*75). Furthermore, categorical data are presented as counts and percentages (n, %). Repeated-measures ANOVA was performed to compare differences in scores among student works across the evaluation dimensions.

## 3 Results

### 3.1 Response and reliability

Pre- and post-questionnaires were completed by 47 and 54 students, respectively. Cronbach’s alpha was 0.73 (pre) and 0.80 (post), indicating good reliability.

### 3.2 Understanding of precision science communication

**Figure 2:**
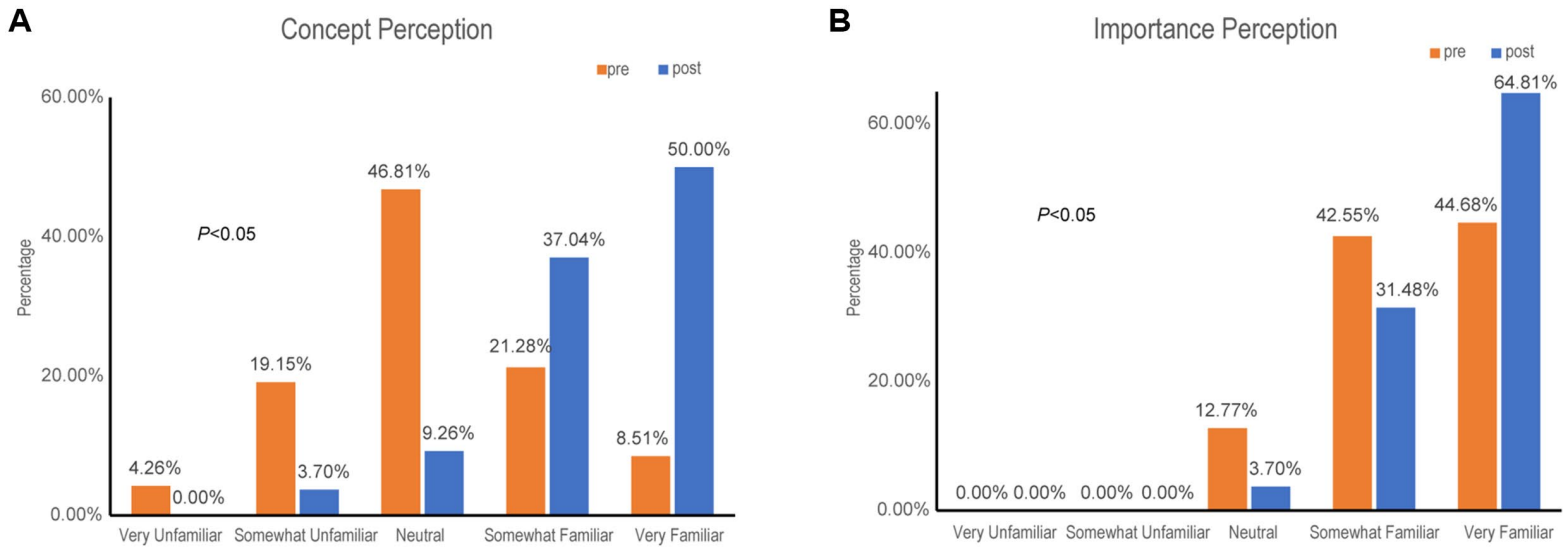
Changes in understanding and perceived importance of science communication. **(A)** Familiarity with precision science communication. Post-course data show a substantial increase in “very familiar” responses. **(B)** Recognition of the importance of science communication. A greater proportion of participants selected “very familiar” after the course. Data are percentages from pre- and post-course surveys.

The proportion of students “very/somewhat unfamiliar” with the concept dropped from 23.41 to 3.7%, while those “very/somewhat familiar” rose from 29.27 to 87.04% (*P* < 0.05) (Figure 2A). Recognition of its importance also increased significantly, with “very familiar” ratings rising from 44.68 to 64.81% (*P* < 0.05) (Figure 2B).

### 3.3 Mastery of precision science communication knowledge

Mann–Whitney U test results indicated a significant improvement in overall knowledge mastery after the intervention (*U* = 995.00, *P* < 0.05). While the median score remained unchanged (*M* = 40), the score distribution shifted upward from an interquartile range (*IQR*) of 30–40 points pre-intervention to 40–50 points post-intervention (Table 1).

**Table 1:** Students’ knowledge test scores on precision science communication before and after the intervention.

Group	Number of participants (n)	Median (*M*)	*P*25, P75	*U-*value	*Z-*value	*P-*value
Pre-intervention	47	40	30,40	995.00	−1.979	*P < 0.05*
Post-intervention	54	40	40,50			

### 3.4 Practical application ability

**Figure 3:**
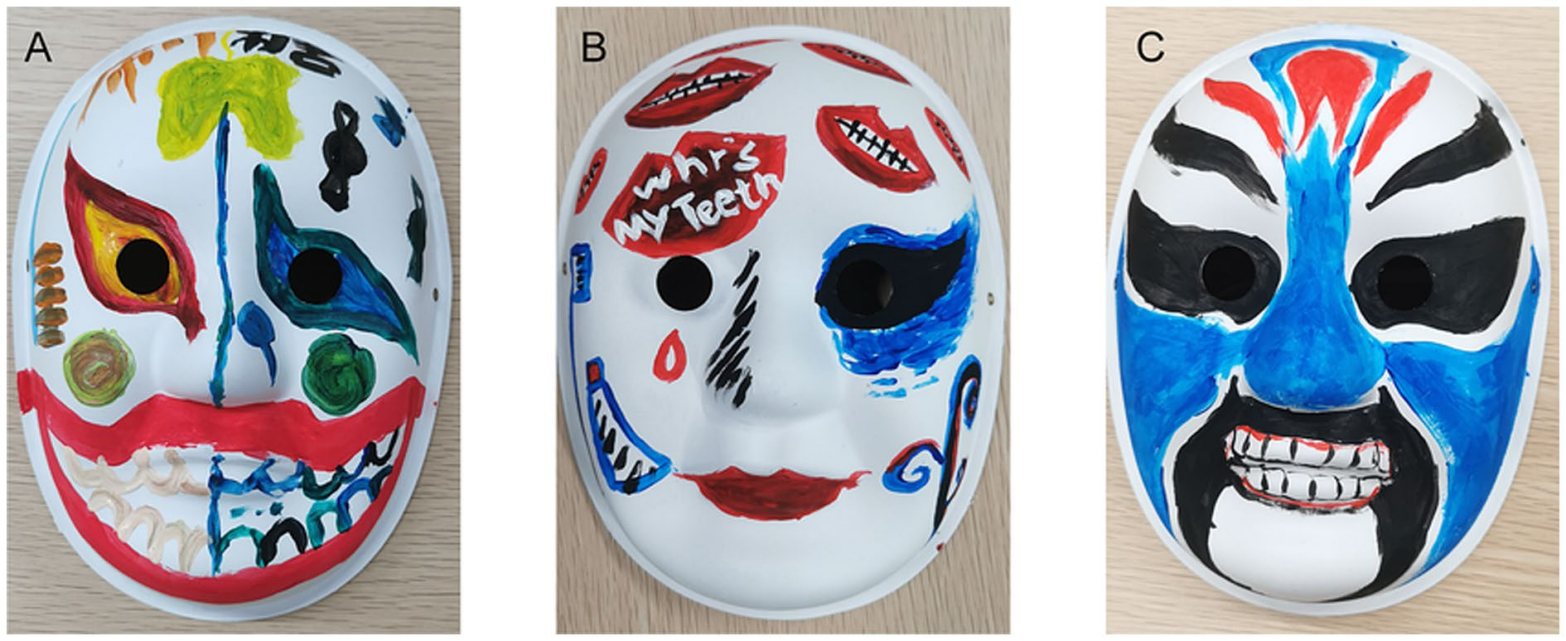
Examples of student-designed science communication works targeting different age groups. **(A)** Group A’s work for adolescents uses a two-sided mask with contrasting images to emphasize oral care habits. **(B)** Group B’s work for young and middle-aged adults employs a red lips design to visually convey toothache mechanisms. **(C)** Group C’s work for older adults integrates traditional opera elements to communicate the importance of tooth restoration.

All three works carefully considered the demographic characteristics, health needs, and cognitive barriers of their respective target audiences. Through differentiated design, they achieved high entertainment value, strong relevance, and notable effectiveness, suggesting the development of demonstrating outstanding practical application abilities. Group A’s work targeted adolescents. One side of the mask depicted healthy teeth resulting from proper brushing, while the other side showed dental stains caused by candy, focusing on the two core needs of “developing a brushing habit from a young age” and “limiting sugar for dental protection.” This comparative facial design, considering the developing learning and comprehension abilities of adolescents, replaced dull lecturing with impactful visual contrast, aiming to translate dental care knowledge into daily behavior (Figure 3A). Group B’s work targeted young and middle-aged adults, using the highly prevalent and painful symptom of “toothache” as the entry point. Leveraging this group’s relatively strong comprehension abilities, it explained the mechanism of pulpitis as nerve pain, helping users understand “why toothache can be so unbearable.” The series of red lips and white teeth created a strong visual symbol, combining fashionable appeal with a health-related metaphor (Figure 3B). Group C’s work targeted the older adult audience, skillfully incorporating elements from traditional opera familiar to this demographic to evoke cultural memory. Addressing the prevalent health issue of high tooth loss rates among this group, the work used clear illustrations of aligned teeth to convey the message of “the importance of paying attention to tooth restoration.” The design fully considered potential comprehension barriers older adults might face when receiving health information, achieving effective communication of professional knowledge (Figure 3C). Peer evaluations are summarized in Table 2. Overall, all three groups achieved favorable ratings across the dimensions of entertainment, relevance, and effectiveness. Notably, Group B, which employed a multiple interdisciplinary composition, attained the highest mean scores in all three criteria: entertainment (9.00 ± 1.30), relevance (9.08 ± 1.11), and effectiveness (9.15 ± 1.00). By comparison, Group A (single-disciplinary, Medicine only) and Group C (dual-disciplinary) showed lower scores across all dimensions, with similar performance levels between the two groups. However, no statistically significant differences were observed between the groups.

**Table 2:** Student peer-evaluation scores for the three science communication works (range: 1–10).

Assessment indicators	Work A	Work B	Work C	*P-*value
Interdisciplinary mix	Single (medicine)	Multiple	Dual	–
Target audience	Adolescents	Young/middle adults	Older adults	–
Entertainment (*M* ± *SD*)	7.23 ± 3.11	9.00 ± 1.30	7.38 ± 3.28	*P>0.05*
Relevance (*M* ± *SD*)	7.38 ± 3.10	9.08 ± 1.11	7.38 ± 3.28	*P>0.05*
Effectiveness (*M* ± *SD*)	7.38 ± 3.10	9.15 ± 1.00	7.38 ± 3.28	*P>0.05*
Suitability	–	–	–	–

### 3.5 Student self-assessment

92.60% of students expressed satisfaction with the A-SPIRE model, with 66.67% reporting they were “very satisfied.” No students indicated dissatisfaction. 96.3% believed their science communication ability improved, and 85.19% expressed willingness to engage in such work in the future (see Table 3).

**Table 3:** Post-course student self-assessment of teaching effectiveness (*n* = 54).

Evaluation dimension	Strongly disagree	Somewhat disagree	Neutral	Somewhat agree	Strongly agree
Satisfied with the teaching model	0(0.00%)	0(0.00%)	4(7.41%)	14(25.93%)	36(66.67%)
Science communication ability improved	1(1.85%)	0(0.00%)	1(1.85%)	24(44.44%)	28(51.85%)
Helpful for future science communication practice	1(1.85%)	0(0.00%)	8(14.81%)	17(31.48%)	28(51.85%)
Willing to attempt precision science communication work in the future	1(1.85%)	1(1.85%)	6(11.11%)	18(33.33%)	28(51.85%)

## 4 Discussion

The findings of this study suggest that the A-SPIRE model may be a promising and actionable framework for integrating audience-tailored public health communication competencies into medical education, with profound implications for aligning medical training with the goals of the “Healthy China” strategy. As a structured, theory-to-practice pedagogical model, it represents a novel contribution to addressing the underdeveloped area of health communication training in current medical education. By guiding medical students through a sequential cycle of concept anchoring, practical application, and iterative evaluation, the model effectively appears to facilitate the translation of abstract health communication theories into tangible skills—aims to address a long-standing gap between theoretical knowledge and clinical-practice needs in medical curricula.

A pivotal finding of this study is the significant improvement in students’ conceptual grasp of precision communication and their ability to value its importance. This advancement is not merely academic; it aligns with foundational health communication theories such as Audience Segmentation theory and the Health Belief Model, which emphasize that effective health promotion hinges on understanding audience-specific needs and barriers (8, 18). The A-SPIRE model’s strength lies in its operationalization of these theories: it moves beyond passive knowledge transmission to create experiential learning opportunities where students must apply theoretical principles to design messages tailored to distinct audiences. This represents another novel contribution of our work: transforming abstract theories into actionable, audience-centered learning tasks. This transition from “knowing” to “doing” is critical, as medical graduates are increasingly expected to act as health educators alongside their clinical roles.

The observed enhancement in both knowledge acquisition (evidenced by upward shifts in knowledge test distribution) and skill transfer (reflected in high-quality student-created artifacts) further provides supporting evidence for the model’s potential utility. Particularly noteworthy is the “Integrating” and “Realizing” stages, which simulate real-world public health campaign development by forcing students to contextualize abstract theories into concrete creative tasks for specific populations. Strong theoretical knowledge is vital for medical students’ communication competence (22). This experiential design addresses a well-documented limitation of traditional medical education—over-reliance on theoretical instruction—by demonstrating that integrating theory with practical application yields more durable communication competencies (23).

A notable observation from the peer evaluations is that Group B, which adopted a fully interdisciplinary composition, achieved numerically higher mean scores across all dimensions of engagement, practicality, and effectiveness, whereas Group A, consisting solely of medical students, showed relatively lower scores. Although intergroup comparisons did not reach statistical significance, this pattern is consistent with our longitudinal observations across the 12-session elective course, in which health communication products developed by groups comprising only medical students consistently exhibited weaker innovation, narrative structure, and visual appeal compared with those created by multidisciplinary teams. This consistent trend, supported by convergent observations from multiple teaching practices, suggests that disciplinary homogeneity may still influence the creativity and diversity of health communication outputs, even if statistical significance was not detected in the present study. Such a pattern may be partially attributed to the relatively homogeneous disciplinary background of medical students and, more importantly, insufficient emphasis on science popularization competencies in current medical curricula, which limits students’ exposure to non-medical perspectives and creative expressions. To strengthen these competencies, we propose two targeted strategies: first, curricular Innovation—introducing specialized courses such as medical visualization, narrative medicine, and communication psychology to build a foundational knowledge; second, practical Integration, establishing multidisciplinary practice platform based on Team-Based Learning (TBL) to foster collaborative problem-solving and cross-domain knowledge integration (24, 25). This approach is supported by transdisciplinary education theory, which posits that co-creation among diverse professionals is key to solving complex public health challenges and elevating the quality of health communication (26–28). Additionally, adopting a transdisciplinary approach that integrates communication studies, behavioral sciences, and AI technical capabilities is crucial for enhancing AI’s competence in health communication (29). By adopting such a model, we aim to enable future physicians to navigate communication barriers and enhance cross-sectoral synergy (30, 31), a competency increasingly demanded in public health practice. For instance, Michael T. Lawless has demonstrated that the provision of practical knowledge is essential for cultivating transdisciplinary collaboration capabilities among professionals at different career stages (32). Similarly, the A-SPIRE model, by incorporating interdisciplinary teamwork, not only enhances student performance but also prepares them for the collaborative nature of modern public health practice.

The high student satisfaction and self-efficacy reported in this study may reflect the model’s long-term potential to shape competent health communicators. This positive reception is partly attributed to the model’s alignment with students’ identified learning needs: 74.22% of medical students have previously acknowledged the need to strengthen core health communication knowledge (11). By building students’ confidence in their communication abilities, the A-SPIRE model contributes to nurturing a physician workforce that is both clinically proficient and capable of engaging communities as trusted health advocates— a core objective of modern public health education.

### 4.1 Limitations and future directions

This study employed a single-group pre-post design, which limits the extent to which the specific effects of the A-SPIRE model can be disentangled from potential confounding variables. Fluctuations in participation between the pre- and post-assessments may introduce self-selection bias. The relatively small sample from a single institution also constrains the generalizability of the findings to wider medical student populations. In addition, the uniform output format (mask design) constrained the diversity of students’ creative expression and the model’s applicability across varied health communication contexts. To overcome these limitations, future studies should incorporate well-defined control groups for rigorous comparative analysis, recruit larger and more representative multi-center samples to improve generalizability, adopt real-name questionnaires or anonymized tracking codes to minimize self-selection bias, and validate the model across diverse output formats such as videos and social media materials.

Beyond these methodological considerations, further research is needed to assess the translational impact and contextual adaptability of the model. Longitudinal tracking is also necessary to examine whether the communication skills acquired in the classroom can be translated into real-world practice. Additionally, integrating direct feedback from target community members—consistent with the community-engaged classroom approach in health communication teaching—would strengthen the evaluation system and further improve students’ practical skills, confidence, and professionalism (33). Some scholars have argued that health promotion practice should prioritize enhancing interaction effects (34), which could be integrated into future iterations of the model (34). Notably, there is currently a lack of a systematic tool for assessing medical students’ health communication competence. In the future, a dedicated scale for evaluating medical students’ health communication capabilities could be jointly developed by medical education experts, public health practitioners, community representatives, and students themselves (35).

Furthermore, in light of ongoing technological evolution, the A-SPIRE framework holds potential for integration with emerging digital tools. Against the backdrop of rapid artificial intelligence (AI) advancement in health communication, the A-SPIRE model also provides a flexible framework for integrating emerging technologies. AI has already transformed health communication by enhancing message immediacy, enabling efficient generation of targeted content, and supporting data-driven strategy optimization (29, 36–38). However, risks such as misinformation, over-reliance on technology, and erosion of interpersonal communication skills necessitate a balanced approach—where AI serves as an adjunct to human expertise rather than a replacement (39). AI is driving digital transformation and reshaping medical education in China (40). Future iterations of the A-SPIRE model could incorporate AI tools (e.g., ChatGPT for message drafting, AI analytics for audience insight) within its practical stages, while emphasizing ethical oversight and human judgment. This integration would address student feedback calling for clearer links between AI and clinical/public health practice (41), and align with the growing consensus that developing AI-enabled communication competence is a key direction for future research.

## 5 Conclusion

Collectively, these findings highlight the capacity of the A-SPIRE model to bridge medical education and public health practice. The novel contributions of this study are twofold: the proposal and validation of the A-SPIRE framework, and the integration of theory-driven experiential learning into health communication education. In an era where the growing burden of non-communicable diseases and persistent health inequities call for proactive and targeted community engagement, cultivating tailored health communication competencies among medical students is no longer discretionary, but imperative. With its structured, student-centered design, the A-SPIRE model serves as a scalable and sustainable approach to enhancing the role of medical education in advancing the Healthy China strategy. Future applications may further explore interdisciplinary integration and adaptability to emerging educational technologies, so as to better align medical training with evolving public health demands.

## Data Availability

The original contributions presented in the study are included in the article/supplementary material, further inquiries can be directed to the corresponding authors.
